# Breaking the explanatory circle

**DOI:** 10.1007/s11098-020-01444-9

**Published:** 2020-03-30

**Authors:** Michael Townsen Hicks

**Affiliations:** University of Birmingham, Birmingham, UK

**Keywords:** Explanation, Laws of nature, Humeanism, Circularity objection, Metaphysics, Grounding, Causation, Metaphysical explanation, Metaphysics of science

## Abstract

Humeans are often accused of positing laws which fail to explain or are involved in explanatory circularity. Here, I will argue that these arguments are confused, but not because of anything to do with Humeanism: rather, they rest on false assumptions about causal explanation. I’ll show how these arguments can be neatly sidestepped if one takes on two plausible commitments which are motivated independently of Humeanism: first, that laws don’t directly feature in scientific explanation (a view defended recently by Ruben in R Inst Philos Suppl 27:95–117, 1990, 10.1017/S1358246100005063 and Skow in Reasons why, Oxford University Press, Oxford, 2016) and second, the view that explanation is contrastive. After outlining and motivating these views, I show how they bear on explanation-based arguments against Humeanism.

## Introduction

Humeans hold that laws of nature are nothing more than true generalizations that play a specific role in our inferential practices. Humeans follow Hume, Mill (1843), Ramsey (1928), and Lewis (1983) in denying necessary connections between distinct events. Since events cannot necessitate other events, laws of nature must be doing something other than enabling or enforcing such necessitation. But without this necessitation, many authors–including Lange (2013, 2016), Emery (2019), and Shumener (2019)–have argued that Humean laws can’t be explanatory. Here, I’ll show that, given plausible assumptions about explanation, these explanation-based arguments fail. The explanatory assumptions I need to show this are well motivated and independent of the debate between the Humean and the nonHumean. If I am correct, careful thinking about explanation shows that the arguments miss their mark, and the Humean need not significantly alter her philosophical account of lawhood, the grounding of laws, or invoke a distinction between scientific and metaphysical explanation.

I’ll set the stage in Sect. 1 by getting clear on the assumptions I need to respond to these arguments, and why I want to. In Sect. 1.1, I’ll introduce and motivate the first of two assumptions I need for my defence: the claim that the laws do not explain their instances, but instead feature in meta-explanations. On this view, the laws explain why some events explain some other events.^1^ This view has recently been defended by Ruben (1990) and Skow (2016), and less recently by Scriven (1962). As this is the more controversial of the two assumptions I’ll need, I’ll attempt briefly to motivate it. Next, in Sect. 1.2, I’ll introduce and discuss the second ingredient in my response to explanation-based arguments against Humeanism: the claim that explanation is contrastive. This is a much more widely held view. Many authors explicitly endorse this view; most leading accounts of explanation imply or at least are compatible with it. Consequently, I will spend less time motivating this view and more time explicating it. Finally, in Sect. 1.3 I’ll provide an all-too-brief overview of the Humean view of laws and explanation. The details of this view don’t matter for the defence I mount later, but I take it to be worth my readers’ while to see what Humeanism is and why it is worth defending.

Having set the stage, I’ll proceed in Sect. 2 to consider two explanation-based objections to Humeanism and show how they run afoul of one or both of my assumptions about explanation: first, that Humean explanation is circular (Sect. 2.1), and second, that Humean laws cannot explain regularities (Sect. 2.2). In the first instance, I’ll show that the circularity argument can be rejected using only the assumptions defended in Sects. 1.1 and 1.2, which (I claim) should be accepted by both Humeans and nonHumeans. I then (Sect. 2.2) argue that regularity-based challenges are directed not just at Humeans, but at anyone who accepts the independently-motivated view in Sect. 1.1; I argue that even on this view nearly every regularity has an explanation, and amongst those few which do not, we can use higher-order explanation to fruitfully delineate the unexplained facts which count against theories.

## Setting the stage

### Laws Don’t Explain

The first of the two assumptions I’ll need is more controversial than the second. According to this view, laws don’t feature directly in causal explanations. Rather, they feature in meta-explanations. According to this view, particular events *e* are fully explained by facts about their causes $c_{1}, \ldots , c_{n}$. The laws are part of the explanation of the fact that these causes *are* causes–which is why they explain *e*. This view, or a view like it, is defended recently and at length in Skow (2016),^2^ as well as Schnieder (2010),^3^ Ruben (1990),^4^ and Scriven (1962).^5^ A brief note: I’ll be arguing that laws do not feature in causal scientific explanations; I don’t mean to commit myself to the claim that all scientific explanation is causal. In fact, in Sect. 2.2, I’ll claim that fundamental laws directly feature in explanations of nonfundamental lawhood; plausibly, these explanations are scientific but not causal.

I don’t have time to fully defend this view here, but I do have space to briefly motivate it, and I think I should. After all, nearly every account of scientific explanation has featured laws as explanans in some way or another. For example, the Deductive Nomological (D-N) Model of Hempel and Oppenheim (1948) explicitly requires explanations to feature general laws amongst the explanans; an explanation is successful only if the explanans include a law which is a necessary part of any derivation of the explanandum. Similarly, the Kairetic model of Strevens (2008) and the interventionist account of Woodward (2003) both require laws or law-like generalizations to feature as explanans in a fully elucidated explanation. Here’s an excerpt from Woodward:

Suppose that M is an explanandum consisting in the statement that some variable Y takes the particular value y. Then an explanans E for M will consist of (a) *a generalization G relating changes in the value(s) of a variable X (where X may itself be a vector or n-tuple of variables* $X_f$) *and changes in Y*, and (b) a statement (of initial or boundary conditions) that the variable X takes the particular value x... (Woodward 2003: 203, emphasis added)

For Woodward, the explanation must include G, an invariant generalization which may or may not be a law: “[m]y argument in this and the following chapter [Chaps. 5 and 6] is that it does not matter, independently of whether a generalization can be used to answer w-questions (or whether it is invariant), whether we decide to classify it as a law or whether it possesses the other features traditionally assigned to laws by philosophers...” (pp. 237-38). To keep things simple I will ignore the distinction between invariant generalizations and laws in the following because I think that the view I defend–and the objections I defend it against–can be recast in terms of invariant generalizations rather than laws.

Similarly Strevens’ Kairetic account takes explanations to be based around causal models, which he takes to be D-N style deductive arguments:

Explanatory information–that is, information about difference-making–is conveyed by a set of causal models that have been, first, stripped down by the Kairetic procedure so as to contain only difference-makers and, then, sewn together to form a standalone explanation. (Strevens 2008: 71)

An atomic causal model of an event will have the same form as a DN explanation of that event [...] Both the causal model and the DN explanation are law-involving deductive arguments that the event occurred... (Strevens 2008: 72)

Neither Woodward nor Strevens advocate a naïve D-N model of explanation; both of them include bells and whistles that allow their views to insure that explanation is asymmetric^6^ and that the all explanans are strictly relevant.^7^ What Woodward and Strevens’ views share with the D-N account is a necessary condition on successful scientific^8^ explanation:

- Law Inclusion Requirement in order for an explanation to be successful it must (a) include laws–or invariant generalizations–amongst the explanans and (b) require these generalizations for a logically or metaphysically valid deduction of the explanandum.

To get a feel for what the Law Inclusion Requirement requires, let’s look at the following purported explanation:

- Ale: This beer is bitter because it was made with lots of hops.

Ale seeks to explain the beer’s flavor in terms of it’s ingredients. Unfortunately, according the the Law Inclusion Requirement, Ale is not even in the running to be an explanation of the beer’s bitterness because it does not include a law linking hops to bitter beer flavor. The full explanation is something like:

- Ale Completed: This beer is bitter because it was made with lots of hops, and all heavily hopped beers are bitter.

Where the fact that heavily hopped beers are bitter is meant to be a law–call it The Ale Law–and is meant to feature in Ale Completed in the same way the adding of the hops does: as an explanans.

The view that Ale Completed requires The Ale Law as an explanans in order for it to be a successful explanation is a widely held view, but it is a very strange view. After all, the laws–even on a Humean account–are very different sorts of things than the other explanans. This is a worry from *ontological variety*. According to this view, there are two quite different sorts of things that a causal or scientific explanation requires: both particular events, which cause the explanandum, and general laws–the things in virtue of which the explanans cause the explanandum. In the Ale case, the fact that the beer contains hops causes it to be bitter; the fact that hops increase bitterness is an explanation for why or how the hops explain the bitterness. The first is a particular fact about this beer; the second is a very general causal fact about flavor and the chemistry of beer production.

That these two sorts of things are quite different should make us suspicious. The general laws are not particular events, they are not so spatiotemporally restricted. They do not occur or fail to occur. They are also not subject to manipulation or control, so, if you are sympathetic to manipulationist accounts of explanation, including the laws amongst the explanans looks quite strange.

The worry from ontological variety leads to another worry, which I think is a bit more interesting. I follow Kim (1994) in thinking that explanations track dependence relations. What’s strange about the Law Inclusion Requirement is that the explanandum depends on the laws in a very different way from the way in which the explanandum depends on its causes. Call this the *mixed-dependence* worry.^9^ An explanandum causally depends on the particular events that are amongst the explanans–those are its temporally antecedent causes. But its dependence on the laws is not causal–the laws are just not the sorts of things that can be causes. The beer is caused to be bitter by the hop addition, not by The Ale Law.

What, then, is the laws’ relationship to the explanandum? This is a bit mysterious. For Hempel and Oppenheim, it is deductive implication. But that is a relationship which holds between statements, not things. Perhaps the laws necessitate the explanandum–but of course, they don’t. They only necessitate the explanandum together with the event-type explanans. But the event type explanans already bear a dependence relation to the explanandum–they cause it. Maybe the the explanandum metaphysically depends on the laws (as in Emery (2019)). But if this is correct, either the explanandum metaphysically depends on its causes (which seems strange) or scientific explanation requires a mixed causal/metaphysical dependence relationship between the explanans and the explanandum. This makes apparently unified explanations an unattractive hodgepodge of distinct dependence relationships.

Finally, the law-inclusion requirement creates a strange mismatch between the sort of explanation and the associated variety of implication. A statement describing some event *c* implies a statement describing *e* just in case the *e*-statement holds at every possible world at which the *c*-statement holds. But what sort of possibility is this? On any model of explanation with the law-inclusion requirement, the relevant sort of possibility is always metaphysical. But this doesn’t allow us to distinguish properly between different sorts of explanation, which correspond to different sorts of necessity. Plausibly, mathematical explanations and purely logical explanations imply their explanandum with mathematical or logical necessity.^10^ Similarly, requiring scientific explanations to imply their explanans with the full force of logical or metaphysical necessity seems extravagant, when nomological necessity is well understood. The fact that the beer is bitter is nomologically necessitated by the fact that it is heavily hopped, all by itself. The Ale Law isn’t required as a premise for a nomologically valid deduction of the explanans. Why require this implication to hold with metaphysical necessity when the explanation is scientific? (The idea that each sort of explanation is associated with its own form of possibility and necessity is defended in Bhogal (forthcoming)).

It’s much more natural, I think, to distinguish between the different ways in which the explanandum depends on its causes and the laws by giving these two different sorts of things with their different sorts of relationships to the explanans different roles to play in our model of explanation. One way to do this, which I find attractive, is to hold that the explanandum must *nomologically* depend on the explanans, where *e* nomologically depends on *c* only if *e* occurs at every nomologically possible world at which *c* occurs. Rather than being logically valid, scientific explanations need merely be nomically valid: the premises must be true at every nomically possible world where the conclusion is true.

If this view is correct, the difference between causes and laws in scientific explanation is akin to the difference between premises and inference rules. If we model nomological explanation as a deduction, then *e* nomologically depends on *c* only if a statement describing *e* can be derived from a statement describing *c* using the laws as inference rules.^11^ Call this necessary condition the *Inference Rule Requirement*:

Inference Rule Requirement: If $c_{1}, \ldots c_{n}$ scientifically explain *e*, then *e* follows from $c_{1}, \ldots , c_{n}$ in a deductive system which includes the laws as inference rules, and at least one law-statement must be essential to the derivation of *e*.

The Inference Rule Requirement replaces the Law-Inclusion Requirement, and retains a special place for the laws in distinctively scientific explanation.^12^

Replacing the Law Inclusion Requirement with the Inference Rule Requirement would leave the content of most models of explanation largely unchanged. All three accounts of explanation mentioned earlier–the interventionist account of Woodward (2003), the Kairetic account of Strevens (2008), and Hempel and Oppenheim’s D-N model–would function in roughly the same way if laws or invariant generalizations were used as principles of inference rather than premises or explanans. But these formalisms would then better represent the relevant structure of causal explanation, in which the explanandum depends causally on the events and this dependence is itself underwritten by the laws. On such a view, the different varieties of dependence are clearly and accurately modelled by the philosophical account of explanation.

But note that on this view, although laws do not feature as explanans, they do, in an important sense, back the explanation. In order for the explanans to explain the explanandum, the two must be connected by at least one law. While hops explain the bitterness directly and by themselves, as in Ale, The Ale Law explains the fact that the hops explain the bitterness, because it is in virtue of this law that the hops nomically entail the bitterness. In general, if we are to explain why some explanans explains some explandum, we will in many cases need to cite a law.

My view is that, by backing these explanations, the laws partially explain the explanatory relation between the explanans and explanandum, and partially ground the causal relationship between cause and effect. The first event explains the second event in part–but not entirely–*because* they are connected by a law. Of course, it is not solely in virtue of the law. What other facts go into determining that *c* explains *e* will depend on the correct account of scientific explanation, a question about which I am maintaining neutrality. But since the correct account of explanation includes the IRR, the explanandum is *explained* by the explanans *partially in virtue of* the fact that the explanandum can be inferred from the explanans via a law. Although the law is not an explanans, it mediates the explanatory relationship, and so grounds the explanation. I take this in-virtue-of or grounding relationship to be a constitutive connection between laws and scientific explanation, and I believe that this grounding is itself a variety of explanation. So I believe that the laws partially explain the fact that the scientific explanation holds.

But not everyone believes this. Some philosophers hold that the laws merely provide evidence or justification for the view that the explanans explain the explanandum. Plausibly this is the view of Ruben (1990) and Scriven (1962). On this view, when we ask why this explains that, we are asking not for an explanation of this explaining that, but instead evidence for the claim that this explains that. The law is such evidence. It’s a reason to accept the explanation but not an explanation itself. I think this view is false, because I think that the law is one of the things that makes the relationship between these events an explanatory one, and I think that such constitutive relationships are explanations. On my view, the law epistemically justifies the claim that there is an explanatory relationship between *e* and *c* because it partially grounds that relationship. Those who deny the grounding claim need an alternative account of the epistemic justification here. Nonetheless, for those who do not agree with me on this, I believe that the arguments I give in Sect. 2 can be rephrased in terms of justificatory reasons rather than higher-order explanation.

Laws, then, regularly feature in higher-order explanations–explanations of explanatory relationships. This leaves us with a view very like that defended by Skow (2016): laws are not themselves reasons why some event occurs, but instead are second-level reasons why the event’s causes produce it.

### Explanatory contrasts

The second feature of explanation I will appeal to in responding to explanation-based objections is the fact that explanation is contrastive. I think that this view is much more widely held, so rather than provide an independent motivation of it, I’ll take this space to say just what I think the contrastivism of explanation amounts to. Contrastivism is implicit in Woodward (2003), where the possible variable settings produce a set of contrasts, and it is at least compatible with most other accounts. See also Dretske (1977a), van Fraassen (1980), Hitchcock (1996), Barnes (1994), Lipton (2004, ch. 3) and Schaffer (2005) for defences of contrastivism.

Contrastivism is the view that both the explanans and explanandum in an explanation include (often unstated) contrast cases. So, for example, when I seek to explain why I’m drinking a Pabst Blue Ribbon (hereafter ‘PBR’), I might in one case cite my preference for the taste of PBR over Heineken; in another context, I might cite the fact that I am thirsty. According to contrastivism about explanation, this is because in the two contexts have different implicit contrasts: in the first case, I’m explaining the (contrastive) fact that I’m drinking PBR rather than Heineken; in the second case I’m explaining the fact that I’m drinking PBR rather than nothing.

Similarly, contrastivism holds that the explanans of an explanation also include often implicit contrasts, and these contrasts are each relevant to producing the contrast cases in the explanandum. So in these cases the fully explicit explanation which includes the relevant contrasts might be something like:

1. I drink PBR rather than Heineken because I prefer the taste of PBR to Heineken, rather than the other way around.
2. I drink PBR rather than nothing because I’m thirsty rather than sated.

An important thing to note here (and this will become relevant later) is that the contrasts in the explanans ought to fit the contrasts in the explanandum. If the explanation takes the general form:

general form: *c* rather than $c'$ explains the fact that *e* rather than $e'$,

then it had better be the case *both* that *c* leads to *e* *and* that $c'$ leads to $e'$. The fact that I’m drinking PBR rather than Heineken isn’t explained by the fact that I prefer PBR to Heineken rather than preferring Schlitz to PBR, because preferring Schlitz would not lead to me drinking Heineken rather than PBR. These contrasts would not fit.

Contrastivism is itself motivated by the idea that explanations are looking for difference makers. The contrasts in the explanandum tell us what we want to make a difference between. The contrasts in the explanandum, then, tightly constrain the contrasts we can have in the explanans. The explanans must show how the difference is made by showing what would have been required for things to be different.

What is it for one event to lead to another here? In light of Sect. 1.1, we should take this to be a form of nomic dependence. Recall that according the IRR, *c* must imply *e* using some law (or counterfactually robust generalization) as an inference rule. It is partially in virtue of this nomic implication that *c* leads to *e*. My claim here is that, similarly, the connection between $c'$ and $e'$ must be backed or explained by a law.

Contrastivism is also motivated as a response to some worries concerning the transitivity of explanation. Often, it seems, explanations can be chained together. If I offer explanation (1) above, and then tell you that I prefer PBR to Heineken because I watched Blue Velvet one too many times, intuitively, it is also the case that I am drinking a PBR because I watched Blue Velvet one too many times. This transitivity relation can be expressed as follows:

naïve transitivity If *a* explains *b*, and *b* explains *c*, then *a* explains *c*.

Naïve transitivity has counterexamples. It seems to license the following inference:

1. I’m a bit tipsy because I had a PBR.
2. I had a PBR because I prefer PBR to Heineken.
3. I’m a bit tipsy because I prefer PBR to Heineken.

This inference looks bad. It looks bad because I would still be tipsy if I’d had a Heineken–the brand of beer doesn’t make a difference. What’s gone wrong here is that we illicitly switched contrasts between P1 and P2: I’m tipsy because I had a PBR *rather than not drinking*, whereas my preference for PBR explains why I had a PBR *rather than a Heineken*.

This leads to the following contrastive transitivity principle:

contrastive transitivity: If *a* rather than $a'$ explains *b* rather than $b'$, and *b* rather than $b'$ explains *c* rather than $c'$, then *a* rather than $a'$ explains *c* rather than $c.'$

This principle eludes the counterexamples (I encourage the reader to check).

Much of the work on contrastivism in explanation has focused on the contrasts in the explanandum, and has been inspired by the connection between explanations and why-questions. van Fraassen (1980) is a key example of this: the contrastivity of explananda is there argued for on the basis of the fact that why-questions are often ambiguous, and this ambiguity can be resolved by explicitly introducing contrasts to the why question. Similarly, Dretske (1977a) argues for contrastivism on the basis of ambiguity in speech acts. In response, some authors–including Humphreys (1989: 137) and Markwick (1999)^13^ have argued that if we look beyond speech act theory, we can find examples of explananda which are not contrastive, and that the focus on acts of explaining distracts from this.^14^

This emphasis on speech acts distracts us from two important reasons to take explanations to be contrastive. First, formally, if we expect explanation to be transitive, then the fact that non-contrastive transitivity principles have counterexamples is a reason to expect explanation to be contrastive. Second, it’s a widely-accepted slogan that explanations look for difference makers. But differences must be between at least two things! The contrastive view of explanation allows us to explicitly state what difference needs to be explained by providing the states that differ. Both of these considerations support general form as the fully explicit explanatory relation.

Contrastivism claims that explanations involve contextually determined and often unstated contrasts; because of the role explanatory context plays in the view, many authors have taken contrastivism to be compatible with epistemic, but not ontic, accounts of explanation (see for example van Fraassen (1980) for arguments of this stripe). The worry is something like this: which contrasts are relevant typically depends on context. But the context depends on the background knowledge of agents, their epistemic interests, and their conversational goals. Since the background knowledge, interests, and goals of agents are not features of objective dependence relations between events, contrast cases are not relevant to the in-the-world dependence relations that explanations supposedly track. Since I just finished a section (Sect. 1.1) where I relied on the idea that explanations are backed by real dependence relations, things now look quite fishy: one of my two ingredients is inspired by an ontic take on explanation, the other by an epistemic take. Nobody will be happy with both.

Thankfully for me, contrastivism about explanation is not only compatible with but (in my view) requires an ontic understanding of explanation. It’s true that the relevant contrast cases are set contextually by the epistemic and conversational interests of agents. But once the contrasts in the explanandum are set, there is an objective fact about what those contrasts depend upon. Our epistemic interests determine whether I should explain why I’m drinking PBR rather than Heineken, or instead explain why I’m drinking PBR rather than not drinking. But once those contrasts are set, epistemic interests are no longer relevant. After we explicitly write out an explanation in the general form above, subjective interests have no role to play. The correct explanans are now determined by the dependence relations that exist in the world. I hold that those dependence relations are backed by the laws, and specifically that, for a causal explanation in general form, the laws explain why *c* leads to *e* and why $c'$ leads to $e'$.

### The humean vision

I now have everything I need to respond to two prominent explanation-based arguments against Humean reductionism about laws of nature. When I do so, my aim will be to show, *irrespective of what account of laws of nature one favors*, these explanation-based worries are misguided. However, since the discussion of these arguments has focused on Humeanism, I think that it’s worthwhile for me to briefly motivate Humeanism, as well as explain why I take Humeanism about laws and explanation to be compatible with the independently-motivated features of explanation I’ve described above. Naturally I hope you find the Humean view I describe compelling, but please bear in mind that the discussion of the arguments I’ll embark on in Sect. 2 relies only on rejecting the law-inclusion requirement and accepting contrastivism.

I think I should start this section by saying what, at the end of the day, I want. I want a beer. An actual beer. I don’t want a merely possible beer; I’ve never had one. And though I’ve tried many types of beer I’ve never run into a counterfactual beer. I am not sure whether I’ve had a necessary beer, and honestly I don’t much care; if I have, it tasted just like the local contingent beer.

The interesting question is, given that I am only interested in actual beer, why do I spend so much of my time thinking about merely possible beers and entertaining counterfactual beers? The answer, presumably, has something to do with connecting me to the actual beers. The power of the Humean view is that if it succeeds it ties the possible and counterfactual beers to the actual ones, and thereby explains why an actual-beer-interested agent like myself spends so much time contemplating nonactual beer.^15^ I think that an account of the metaphysics of laws ought to pass the actual beer test–it should help explain why an investigation into counterfactual, possible, and necessary beers is relevant to my pursuit of actual beer.^16^

These modal notions are interconnected: laws, causation, counterfactuals, and explanation form a interrelated package of modal machinery which we employ when devising strategies to achieve our ends. The traditional Humean picture of how this goes is as follows: regularities in nature are summarized in laws. These laws are held fixed when we consider alternative courses of action, and so are counterfactually stable. Causation and explanation both use counterfactuals to determine which events made the difference for the occurrence of some target event, given the laws.

The traditional Humean view of laws is that handed down through Mill (1843), Ramsey (1928), and Lewis (1983). According to this view, laws are those general statements which together form a simple, highly informative axiomatic system of all the truths in the world. Our interest in them boils down to our interest in making true, deductive inferences. Recently, a number of Humeans have added more pragmatic considerations to this recipe. In addition to simplicity and informativeness, theorists like Dorst (2019), Hicks (2018), and Jaag and Loew (2018) argue that the features that pick the laws out of the truths of the world include things which make them useful to limited agents like us. Hicks (2018), whose version of the view I understand best, focuses on wide applicability and informativeness concerning particular subsystems of the universe. These features are supported on broadly pragmatic grounds: they’re features of generalizations that make them available for inductive discovery and enable them to produce predictions concerning the small-scale bits of the universe in which agents act. For these Humeans, the laws are those generalizations which are best suited to be discovered by embedded agents observing one small part of the world and applied by those agents when operating in other parts of the world.

How does this account of laws connect to explanation? A standard view of explanation holds that explanation is closely related to manipulation: in seeking an explanation of some Q, we look for those antecedent facts from which Q follows. For the Humean, the ‘follows’ here really is just a form of idealized inference: if the laws and P together determine Q, this just amounts to the fact that Q could have been inferred from P using the laws. But we know that in real cases there are impractically many things antecedent to Q on which Q depends in this way; so we employ what Strevens (2008) calls a “optimizing procedure” to identify the most salient antecedent events on which Q depends: those without which, had other things remained constant, Q would not follow. These are sometimes called ‘minimal difference-makers.’

It’s not hard to see why a beer-interested agent would be motivated to engage in this sort of practice. By finding the minimal difference-makers, we find those things which–given our best tools for prediction–will lead to beer with minimal effort or expenditure in similar situations. It is these which we use in practical decision situations to bring about our desired end. On this understanding of the role of explanation, an explanation helps us by identifying those factors which, in similar situations, can be manipulated to bring about our desired ends.^17^

This story makes sense of the fact that causation plays a central role in both explanation and decision making. When we provide an explanation of an event, we list its difference makers. We can then abstract away from this explanation and come to causal principles. These causal principles can then be used to make decisions and achieve our goals. Explanations, on the Humean view, seek to find antecedent events which are such that, if we bring about similar events, we will get similar results. Laws, on this view, are general principles telling us which respects of similarity matter.^18^ So laws can be used to find both explainers and the sorts of events which will bring about our goals. But laws are not themselves (first-order) explainers or the sorts of events which we can use to bring about our goals, instead, they are abstract principles which link them. We learn what the laws are by abstracting away from a number of first-order explanations; we employ them to make decisions and achieve our goals.

In this story about a beer-interested agent’s use for explanation in a Humean world, the laws and the antecedent events play importantly different roles. The antecedent events are those things that the agent lines herself up with or produces in order to achieve her desired beer. The laws are an inferential tool that help her identify these handles for manipulation. Many accounts of explanation, including Strevens’, include both roles in the story of what first-order explanation is. But if we are thinking, as we are here, of explanations as attempts to find the handles that enable us to reach our aims, then it’s important to separate these roles. The antecedent events are tools we use to produce these ends, and so these explain the later events. The laws cannot be manipulated, and so are not tools for achieving our ends. Instead, they are tools for finding the things that produce the ends; their role is not to explain the events themselves, but the relationship between them.

While this story about the role of explanation is a Humean one, I think there is much here for nonHumeans to like. For nonHumeans, at least as much as Humeans, see our epistemic and practical relationship to the laws as different from our relationship to particular events. For the nonHumean, the laws are out of reach not just as a matter of fact but as a matter of metaphysical categorization. They’re just the wrong sorts of things to be causes, and the wrong sorts of things to be the subject of our action. So a nonHumean who likes the manipulationist account of the practical utility of explanation but dislikes the Humean account of laws should nonetheless buy into the distinction, and build it into her account of explanation. While well-motivated by Humeanism, the theses presented in Sects. 1.1 and 1.2 ought to be common ground in this debate–even though, as I’ll show below, they undercut a common argument against Humeanism.

## Consequences for Humeanism

I think that the Humean view sketched here provides us with the resources to explain our interest in the possible despite the fact that our desires and experiences are situated in the actual. But many a philosopher doubts that the vast space of possibilities can be reached in so simple a ship. In this section, I’ll consider two recent challenges to the Humean view. In Sect. 2.1, I respond to the claim that Humean explanation is circular. In Sect. 2.2, I address the worry that the regularities themselves call out for an explanation that those who deny the Law Inclusion Requirement cannot give.

### The circularity objection

Accusations of circularity have dogged Humeans at least since Dretske (1977b); most recently these have been advanced by Lange (2013, 2016), as well as Maudlin (2007: 172) and Bird (2007). Meanwhile Shumener (2019) and Roski (2018) have offered strengthened versions of this accusation. The circularity argument typically goes like this: (P1) the fact that *L* is a Humean law is explained by the totality of particular facts. But, on standard views, (P2) laws explain those facts! So, by the transitivity of explanation, (C) the facts explain themselves. Humean responses which grant the second premise–that laws explain the facts–can be found in Loewer (2012), Hicks and van Elswyk (2015), Marshall (2015), and Miller (2015).

For reasons given in Sect. 1.1 and suggested in Roberts (MS) and Dennison (2013), this argument doesn’t touch the Humean view presented here: this Humean denies that laws explain the facts, and for independently-motivated reasons. The argument in Sect. 1.1 doesn’t mention Humeanism, and the view presented in Sect. 1.3 cleanly explicates the relevance of unexplanatory laws to agents in a Humean world. So the Humean can and should deny the second premise. There is no circularity, because the laws don’t explain the facts at all. (Note that this response improves upon those of previous authors by dodging Roski (2018) and Shumener (2019)’s semantic account of circularity).

Nonetheless, Lange and others remain suspicious that some sort of circularity remains. Lange (2013: 258) addresses this response in a footnote, and Lange (2016) compares this move to one discussed by Wesley Salmon (1967) in response to the problem of induction. Salmon considers a cheeky interlocutor who uses inductive reasoning to infer that induction works, but never assumes the principle of uniformity of nature. Salmon claims that this reasoning is nonetheless circular, because although it doesn’t use the conclusion as a premise, it does rely on the conclusion in making the inference. Lange writes:

I suggest that likewise, there are two ways for a purported scientific explanation to fail because of circularity:

(i′): by using *p* in the explanans in an explanation of *p*, or
(ii′): by using *p* to help explain why (if *p* obtains) a given *q* can serve as part of the explanans in an explanation of *p*.

(Lange (2016))

This leads to a revised circularity argument (minding our *c*’s and *e*’s rather than *p*’s and *q*’s to retain consistency with Sect. 1.1):

revised circularity argument

1. An explanation is problematically circular if it uses *e* to help explain why (if *e* obtains) a given *c* can serve as part of the explanans in an explanation of *e*.
2. If the Inference Rule Requirement is true, then the laws explain why (if *e* obtains) a given *c* can serve as part of the explanans in an explanation of *e*.
3. If the laws are Humean, then *e* helps explain why the laws are what they are.
4. If the laws are Humean, and the Inference Rule Requirement is true, then *e* to helps explain why (if *e* obtains) a given *c* can serve as part of the explanans in an explanation of *e* (from P2 and P3 via the transitivity of explanation).
5. If the Inference Rule Requirement holds, and the laws are Humean, the explanation of *e* is problematically circular (from P1 and IC).

Here, I believe that the inference to the intermediate conclusion fails. It fails because, as I discussed in Sect. 1.2, the correct transitivity principle is a contrastive principle. P2 and P3 do not have the same contrasts, so they cannot lead to IC. Importantly, the fact that they do not have the same contrasts does not rest in any way on Humeanism about laws, or any sort of distinction between different sorts of explanation.^19^

To figure out what contrasts feature in the explanans of the second-order explanation here, we need to first figure out what implicit contrasts are involved in the explanandum. We need to put the explanation in the general contrastive form of Sect. 1.2. The target of our meta-explanation is, Lange puts it^20^ (moderately paraphrased): why (if *e* obtains) a given *c* can serve as part of the explanans in an explanation of *e*. The relevant contrast case is one in which *c* *cannot* serve as part of the explanans of *e*. The explanandum here is that *c* explains *e*, *rather than not explaining it*.

Once the explanandum contrasts are explicitly stated, it is much easier to see what the explanans contrasts will be. The fact that *c* explains *e* rather than not explaining it is itself explained by the fact that the connection between *c* and *e* is lawful rather than accidental. If it had been an accident that *c* and *e*, then *c* would not explain *e*. In the general form of Sect. 1.2:

Explanatory Claim: The fact that *if* *c* *then* *e* *is an instance of a law* rather than *accidental* explains the fact that *c* *explains* *e* rather than *not* *explaining* *e*.^21^

The important thing here is that neither of the explanandum contrasts are cases in which *e* does not occur. So both of the explanans contrasts will also be cases in which *e* occurs. So–*no matter what view of laws you have*–the fact that *e* occurred rather than did not occur will simply not be part of the explanation that *c* explains *e* rather than not explaining it.^22^

Now, what your view of laws must do next is explain why *if* *c* *then* *e* is an instance of a law rather than an accident. But note that if *c* and *e* are accidentally, rather than lawfully connected, they still both occur.^23^ The occurrence of *e* will not be a difference maker here.

Different views of laws have different explanations of why *if c then e* is a law rather than an accident. On a nonHumean view, this will be because *if c then e* is backed by your favorite nonHumean whatnots. On a Humean view, *if c then e* is a law rather than an accident because it features in the best system, rather than not featuring in the best system. But note that in order for the relevant contrasts to line up, and so for transitivity to work, the contrast cases–in which *if c then e* is not a part of the best system–will still be cases in which *if c then e* is true. If it wasn’t true, it wouldn’t be an accident. Of course, on both Humean and nonHumean views, one way for *if c then e* to fail to be a law is for it to be false. But that way is not relevant to the explanation of *c*’s explaining *e*.

I’d also like to point out here that nothing I’ve said here relies on the dubious counterfactual claim that if *if c then e* had not been a law, it would still have been true. I do not know whether this counterfactual is true and I don’t care–it’s simply irrelevant to the evaluation of the relevant contrast cases. The explanans contrasts here are fixed by the explanandum contrasts, not by counterfactuals solely concerned with the explanans. Thus the question we’re concerned about is not whether *if c then e* had not been a law, would it have been true. Rather, we are wondering whether had it been accidental, it would have been true. This is the question guided by the contrast in the explanandum. And the obvious answer is that yes, it would have been accidentally true.

My guess is that at this point some readers will suspect that I have chosen my contrast cases too carefully. I do not think that I have done so; the explanadum contrasts are the ones that seem natural for the premises in the revised circularity argument above. Go back and check! However, I can imagine a creative interlocutor coming up with a few alternatives. Here are some I’ve thought of on her behalf:

Why, for example, don’t we explain the fact that *c* explains *e* rather than explaining something else? The answer is that the contrasts must be in some way opposed to one another. But it is compatible with *c* explaining *e* that it also explains other things.

Perhaps we should explain why *c* explains *e* rather than explaining $e'$, where $e'$ is the alternative we originally gave for *e*. By stipulation, $e'$ did not occur. And in fact, *c* rather than $c'$ explains the fact that *e* occurred rather than $e'$. Why does *c* rather than $c'$ not instead explain $e'$ rather than *e*? Surely the fact that *e* occurs is relevant to the explanation of this fact!

I agree that the fact that *e* rather than $e'$ occurred is relevant to the explanation of the fact that *c* rather than $c'$ doesn’t explain that $e'$ rather than *e*. In fact, I think it is the complete explanation of the fact that *c* doesn’t explain $e'$. If something doesn’t occur, it cannot be explained. To revisit the example in Sect. 1.2, we are being asked to explain why my preferring PBR explains the fact that I drank PBR, rather than explaining the fact that I drank a Heineken. Since I didn’t drink a Heineken, the fact that I prefer PBR couldn’t possibly explain that I did. Nothing can.

Of course, the *non-occurrence* of $e'$ has an interesting explanation. That explanation is that *c*, rather than $c'$, occurred. As we’ve already agreed, the fact that I prefer PBR explains the fact that I didn’t drink a Heineken. If $c'$ had occurred, $e'$ would have had an explanation. But since it did not occur, it does not require an explanation. Nothing about the laws needs to be involved here.

Perhaps we should explain why *e* is explained by *c*, rather than not occurring. This is a strange thing to someone to explain. Imagine I asked you why you ordered a PBR, rather than never existing. I imagine that you would have a response to this, but that response would involve two distinct explanations: one explanation for your existing rather than not existing, and another for your ordering a PBR rather than something else. Not every putative pair of contrasts has an interesting or unified explanation.

Similarly, to explain why *e* is explained by *c*, rather than not occurring, we have to give two explanations. The first will be an explanation of *e*’s occurrence rather than nonoccurrence. But as we’ve discussed, the explanation of that will be *c*’s occurrence. Then we will need an explanation for *c*’s explaining *e*. But this will also be the explanation we gave before, and it will presuppose that *e* occurs (after all, we’ve already explained that *e* occurs by citing *c*.)

Perhaps the suspicious reader thinks we should explain why *things like* *c* cause, explain, or produce *things like* *e*. Now, perhaps, the explanation *will* involve *e*, at least if Humeanism is true (Although I think often it won’t–I’ll discuss these sorts of explanations in more depth in Sect. 2.2). But even if *e* is involved, no worries: the fact that things like *c* cause, explain, or produce things like *e* isn’t part of the explanation of *c*’s explaining *e* (rather than not). The fact that *c* explains *e*, recall, is explained by the lawhood, rather than accidenthood, of if *c* then *e*, not the distinct fact that *c*’s usually produce *e*’s, so no circularity can arise. The subject has shifted.

I’m confident that a sufficiently suspicious reader will find other potential contrasts for P2 in the argument above. But not all readers are so suspicious, and I worry that if I continue down this path more trusting readers will lose interest and stop following. I hope that the examples given above illustrate my strategy for responding to similar attempts to choose troublesome contrasts.

### Explaining regularities

The circularity arguments is one of the most pressing and compelling explanation-based arguments against Humeanism. Since it is widely agreed that self-explanation and circular explanations are highly problematic, a sound argument that Humeans are committed to such explanations would be devastating for the view. I hope now to have shown that no such sound argument exists. Sadly, though, the circularity argument is not the only explanation-based argument against Humeanism. Although the primary purpose of this paper is to respond to the circularity argument, I will now take some time to show that the assumptions of Sect. 1 shift the playing field for other objections to Humeanism. I don’t think these principles provide so decisive a refutation to these arguments, but I also think that the central premise of these other objections is not as well-supported as the central assumptions of the circularity argument.

Emery (2017, 2019) and Lange (2018: 1350) present such a difficulty: according to them, leaving robust regularities unexplained violates scientific methodology. These regularities, according to Emery and Lange, are explained by the laws.

What, exactly, is this norm on scientific practice? Emery (2017: 489) calls it the pattern-explanation constraint:

The pattern-explanation constraint: Insofar as the only way to avoid leaving a robust pattern unexplained is to introduce a type of entity that is metaphysically weird or novel, we ought to introduce such entities.

What is a “robust” pattern? According to Emery, it is “a pattern that holds under a variety of temporal, spatial, and counterfactual conditions” (Emery 2017: 484). Emery (2019) argues that science ought to explain facts like

1. Why was the event of applying a force of *f* to a mass of *m* at $t_1$ followed by an acceleration of $a = \frac{f}{m}$ at $t_2$ in the many experiments you observed?

Emery thinks that the only explanation of these regularities can be a law, and so, in keeping with scientific practice, we must posit laws to explain them–even if those laws are quite weird things.

Lange similarly holds that Humeans cannot meet an important explanatory burden. After noting that the view allows Humeans to dodge the sort of circularity argument offered above, Lange says:

However, suppose we shift the explanandum from Ga to the fact that all F’s are G. It seems that in scientific practice, an explanation of the fact that all F’s are G can be that it is a fundamental law that all F’s are G. But if the fact that p is a fundamental law does not help to explain why Ga, it seems that by the same token, the fact that p is a fundamental law does not help to explain why p. In that event (by contrast to the case where Ga is the explanandum and Fa is the explanans) there is nothing to serve as the explanans! (Lange 2018: 1350).

Lange and Emery argue that scientific practice requires an explanation of robust regularities, and that the Humean cannot explain these. But of course their argument is not against Humeanism, but instead attacks any view which rejects the Law Inclusion Requirement and incorporates the Inference Rule Requirement, because these views hold that laws are not directly included in explanations. Recall that the shift from the Law Inclusion Requirement to the Inference Rule Requirement was motivated on grounds independent of the debate between Humeans and non-Humeans–in fact, some of our reasons for making this move look stronger on nonHumean than on Humean views. This shifts the debate from a focus on Humeanism to one which focuses on the role laws play in explanation. Do those who reject the Law Inclusion Requirement violate scientific methodology?

I think that the strategy employed in Sect. 2.1 can be used to show that there is no unmet explanatory burden. In some cases, the regularity is non-circularly explained by a law; in others, it is explained, but not by a law. In those rare cases in which there are no available explainers, it is nontheless neither a mystery nor an accident, and so not a cost to the theory.

To figure out whether laws features in the explanation of robust regularities, we should first ask what contrastive fact is being explained. Are we meant to explain why these regularities are robust (in Emery’s sense) rather than flimsy? If so, this fact will obviously be explained by the laws on either Humean or nonHumean views.^24^ The regularity is robust rather than flimsy because it is a law rather than an accident. Many important explanations in science are explanations of this sort. For example, there is an interesting explanation from Newtonian gravitational theory for the fact that elliptical orbits are stable rather than not. The stability of the orbits is a counterfactual fact: the orbits are stable in the sense that small changes in their initial conditions *would* not result in the orbit collapsing. The laws directly explain this stability and thereby the counterfactual robustness (rather than counterfactual fragility) of the fact that planets orbit in ellipses. Similarly, the laws will feature in an explanation of the fact that all stable orbits are ellipses rather than, for example, squares. It is the stability of elliptical orbits and the fragility of non-elliptical orbits that the laws explain. As before, both Humeans and non-Humeans typically accept that counterfactuals are grounded in the laws, and so that the laws are suitable to explain counterfactual facts. The disagreement between the views concerns how that explanation goes.

Does the fact that the orbits are in fact elliptical, rather than not, feature in this explanation? Here, as before, the answer is no. Because the contrastive explanandum presupposes the truth of the regularity, its truth will not be a difference-maker for their robustness. Just as before, the difference-maker cannot be that the orbits are in fact elliptical (rather than not), because this is presupposed by both contrasts in the explanandum–which was that the orbits are stable rather than flimsy.

Should we instead explain why it’s true, rather than false, that planets orbit in ellipses? I agree that general facts like this one call out for explanation. But I am not convinced that rejecting the Law Inclusion Requirement prevents us from giving this explanation. This would only hold if, as Emery and Lange presuppose, the only explanation could be that it’s a law that planets orbit in ellipses. But for the vast majority of robust, non-accidental generalizations, it’s just false that there is no explanation of their truth other than their lawhood–even on accounts of explanation that include laws as explanans. And it is not scientific practice to, when faced by such robust facts, explain them simply by stating that they are laws.

To see why we should look at accounts of regularity explanation. How do standard accounts of causal explanation deal with the explanation of patterns or generalizations? Strevens (2008: ch. 7) treats regularity explanation in the same way he treats explanations of particulars. Strevens uses as an example of a non-accidental regularity the fact that *All ravens are black*. “The mechanistic approach to regularity explanation proposes to explain such a law by exhibiting the causal mechanism in virtue of which the connection exists.” But, Strevens argues, this explanation will not feature only laws. After proposing a causal model which blackness is explained by “natural conditions, the physiological properties of ravens, and biochemical laws,” Strevens asks “Is this causal model sufficient to explain raven blackness? No. It is sufficient to explain why, in natural conditions, anything with [the physiological properties] is black, but in order to explain the blackness of ravens, it must be supplemented with a statement of the form *All normal ravens have [the physiological properties].* This is what I call a *basing generalization*.” (Strevens 2008: p. 229). Strevens goes on to argue that basing generalizations are ubiquitous in regularity explanation.

Strevens’ account of causal regularity explanation is a parallel to his account of event explanation and, as I argued in Sect. 1.1, the Inference Rule Requirement should replace the Law Inclusion Requirement in precisely the same way. The fact that all ravens are black is explained, not by the biochemical laws, but by the basing generalization, and the holding of “natural” background conditions. The biochemical laws, on this view, explain the fact that the physiological properties explain the blackness. The laws are not explanans in this explanation, but ground or back the explanation.

Nearly all regularity explanations require information other than laws, including both basing generalizations and specification of natural conditions, in their as explanans. So, nearly all regularities have an explanation even if the Law Inclusion Requirement fails. The explanans of these explanations are the basing generalizations and natural conditions. For example, the fact that all planets in the solar system in fact orbit in ellipses is explained by the fact that dust cloud that became the solar system had a nonzero net angular velocity and that the velocities of the particles in it did not have statistically unusual correlations. The laws of gravity explain the explanatory fact that this basing generalization explains the resultant elliptical orbits.

This concludes my main response to Emery and Lange: the response is that we do not violate scientific methodology by rejecting the Law Inclusion Requirement. Explanations of robust patterns can still be given, both of their truth and of their robustness, without circularly invoking Humean laws as explanans.

That said, a very small number of regularity explanations do not require these non-nomic facts, and follow directly from the fundamental laws. These include most prominently the content of the laws themselves: what explains, for example, the fact that massive bodies gravitationally attract one another?

If one was asked this question, the natural way to respond would be to show how this fact follows directly from the laws, or just to present the relevant force law.^25^ But if I was right in Sect. 1.1, the law cited can’t explain the regularity (whether or not Humeanism holds). What, then, is going in this apparent explanation?

On my view of explanation, which incorporates the Inference Rule Requirement to replace the Law Inclusion Requirement, this amounts to a meta-explanation. It is an explanation of the fact that gravitational attraction is not an accident. But it shows this without presenting any explanans for gravitational attraction: it shows that gravitational attraction is a nomic consequence of anything, and no explanans are necessary to explain it. This is an explanation of the lack of explanans.

This meta-explanation gives us grounds to distinguish between two ways a pattern can lack explanation. One way is by being inexplicable; another is by nomically following from any event whatsoever. In the first case–the accidental case–there are no potential explanans from which the explanandum follows, via the laws. In the second case, the explanandum follows from any potential explanans via the laws, and so no particular set of explanans meets the other requirements standardly placed on explanation, which remove irrelevant information from the explanation. Although there are plenty of things which nomically imply the explanandum, our optimizing procedure removes all of them. When these generalizations are mere accidents, they are a cost of the theory. When they follow trivially from the laws–with no explanans required–they are not.

I think that making this distinction is enough to respond to the claim that Humeanism violates scientific practice by leaving robust patterns unexplained. But the proponent of the Inference Rule Requirement can, if she wishes, go a step further. Depending on her full model of explanation, she could regard these generalizations as special cases of scientific explanation. If she does, she would thereby reject a widely-held view on explanation:

- Explanation=explanans: If an event or regularity is explained, then some event or fact explains it.

Explanation=explanans is intuitively plausible: after all, most acts of explanation consist in providing or stating the explanans for an event. If there are no explanans, it obviously impossible to give an explanation in this way.

If we reject Explanations=explanans, we could hold these generalizations are explained, but not explained by any facts or events. They are null explained–explained by the null set of premises. On this view, facts that follow directly from the laws are null explained, whereas accidental facts are not like this–they are unexplained. The difference between null explained facts and unexplained facts is an important one: unexplained facts are a cost for a theory; null explained facts are not.

Is null explanation *really* explanation? I am on the fence. Explanations, on a view which includes the Inference Rule Requirement, consist in sentences which imply the explanandum when the laws of nature are employed as inference rules, such that this derivation meets certain asymmetry and minimality constraints. It’s not my aim here to discuss what those asymmetry and minimality constraints are. But it seems to me that they may well be met by a null explanation; null explanation seems particularly well suited to meet minimality constraints, and it’s hard to see how such an explanation could be symmetrical. Consequently I lean towards the view that these fundamental generalizations *are* explained, even though there are no explanans in the explanation.

Grounding theorists make a similar distinction for tautologous sentences: Fine (2012) suggests that some statements, like certain necessary truths and conjunctions without conjuncts, might be ‘zero grounded’: “in the special case in which the operator $\wedge$ was applied to zero statements, the resulting conjunction $y = \wedge ()$ would be grounded in its zero conjuncts. Indeed, the case of zero-grounding may be more than an exotic possibility...” It does not seem to me that null scientific explanation is perfectly analogous to zero-grounding: for example, not every case in which an explanandum follows trivially from a grounding base is a case of zero-grounding (P or not P, for example, is not zero grounded but instead grounded by whichever disjunct holds^26^). But the notion of grounding without grounders is similar to the notion of explanation without explanans.

Whether or not we’re willing to accept explanations without explanans, Humeans do not have an unmet explanatory burden. The explanatory requirement posited by Emery and Lange attacks not Humeanism, but the independently-motivated view that explanations need not include laws. But those of us who deny the Law Inclusion Requirement can provide explanations for nearly every interesting pattern, and can easily distinguish between those unexplained patterns which are an explanatory burden on a theory and those which are not.

## Conclusion

Humeanism about laws is beset by worries about the law’s explanatory power. Here, I’ve argued that if these arguments are examined carefully, they fall apart–not because they misconstrue the Humean explanatory picture, but because they are insufficiently explicit about the structure of explanation and the targets of scientific explanation.

I think that nonHumeans will remain disappointed by the view here. In looking for explanation from the laws, many nonHumeans are seeking what Beebee (2006) calls the “metaphysical glue” that holds the world together. Humeans deny that there is any glue, and so can’t make it out of their laws. What I’ve argued for here is that scientific explanation doesn’t require such glue. Now, I intend to have a beer.

## Footnotes

1 Is explanation a relationship between facts, between statements, or between things? I think that it is a relation between facts which entails a dependence between the things those facts involve. Statements of course are relevant insofar as they describe or represent facts. So, I am committed to an ontic, rather than epistemic, account of explanation. I don’t think that the distinction between facts, things, and statements will matter much for my arguments, but the requirement that explanations describe objective dependence relations will.

2 Skow quite explicitly does not discuss explanation, but instead focuses on answers to why-questions which he takes to be reasons. In discussing an example involving a falling rock (Skow 2016: 75) Skow says “the fact that the rock was dropped from one meter is offered as a reason why it hit the ground at 4.4 m/s, while the law that $s=\sqrt{2dg}$ is offered as a second level reason why, a reason why the drop height is a reason why the impact speed is 4.4 m/s. The law shows up in the answer to the second-level why question, not in the answer to the first level one.” I believe that everything I say here can be recast as a discussion of reasons why in Skow’s sense, but as I aim to respond to arguments which are cast in terms of explanation I will continue to use that terminology.

3 “A causal explanation need not *mention* causality in any way [...] what makes something a causal explanation, I suggest, is not that it is a fact about causes but that it is *grounded* in such a fact” Schneider (2010:327-8).

4 Ruben (1990: 105) argues that laws are unnecessary in explanation because the causes of the explanandum are sufficient for it: “in a singular explanation that is sufficiently full, there may simply be no work that remains for a law to do by its inclusion in the explanation.” Later he claims (p. 106) that if o’s being F explains its being G, “all of the explanatorily relevant properties of o [e.g. o’s being F] can be cited without inclusion of any law or lawlike generalization.”

5 For Scriven, laws are often not a part of an explanation, but the justification for taking some facts to explain some other facts: “[w]hy, then, should one suppose that our grounds for (believing ourselves justified in putting forward) a particular explanation [...] are part of the explanation? They might indeed be produced as part of a *justification* of (the claim that what has been produced is) the explanation. But surely an explanation does not have to contain its own justification [...] Yet, the deductive model of explanation requires that an explanation include what are often nothing but the grounds for the explanation” (Scriven 1962: 67).

6 Thereby avoiding flagpole/shadow counterexamples, in which the fact that the flagpole’s shadow is 3 feet long “explains” the fact that it is 9 feet tall.

7 Thereby avoiding hexed salt counterexamples, in which the fact that this is bewitched salt “explains” the fact that it dissolves.

8 This is slightly misleading: while including a law is necessary for a D-N explanation, the D-N account itself is silent about whether there are non-D-N explanations, and Hempel clearly accepted explanations without laws–for example statistical explanations. Nonetheless, the D-N model takes the inclusion of a law as a necessary condition on D-N explanations, and the other models of explanations discussed likewise require laws to be explanans for an explanation to of the type they model. Thanks for an anonymous reviewer for discussion of this point.

9 This worry is very clearly expressed in Jaag (MS).

10 By claiming that explanations can be backed by different dependence relations, and that these dependence relations are associated with characteristic modalities, I don’t mean to claim that ‘explanation’ is ambiguous, any more than pointing out that there are different sorts of birds commits me to the claim that ‘bird’ is ambiguous. There is just one relation here, the explanation relation. Nonetheless, there are important and interesting differences between some instances of this relation; one difference, I hold, is that while all relations are backed by dependence relations, and all explanans are modally related to their explanadum, there are different forms of dependence and different types of modality. ‘Dependence’ and ‘necessity’ are univocal despite there being philosophically fruitful subdivisions of necessity and dependency; explanation, which is connected to necessity and depency, is similarly a univocal concept despite being similarly susceptible to fruitful subdivision.

11 What does it mean to use the laws as inference rules? Here I just mean that the laws can be relied on in deriving a conclusion, but need not be thought of as premises or be explicitly stated. For example, many laws are stateable as mathematical identities. The acceleration of an object is equal to the net force applied to that object divided by its mass. A system which takes this law to be an inference rule would allow, as an inferential step, the substitution of force divided by mass for acceleration. In this system an argument from premises stating the gravitational force on an object and its mass to a conclusion about its acceleration or future position would be valid without explicitly stating the law. I don’t mean to imply that the laws are not truth apt, or are not descriptive, and instead are merely habits or expressions of hypothetical inferences; just that they need not be explicitly assumed as premises in order to enable inferences in scientific explanations.

12 Note well that the IRR is not meant to be a full account of explanation, and that I do not here intend to offer a full account of explanation. Rather, IRR is meant to replace the Law-Inclusion Requirement in accounts of explanation which feature the Law-Inclusion Requirement, including manipulationist accounts like that in Woodward (2003) or Strevens’ (2008) Kairetic account.

13 Humphreys, for example, argues that the difference between ‘why did the *flame* burn green’ and ‘why did the flame burn *green*’ need not be cashed out in comparison to the flame burning red. Markwick agrees, and argues that such examples show that not all explanations are of contrastive facts. I am not convinced; the difference may well be between an explanation of the flame’s burning green *rather than some other color* and the flame’s burning green *rather than something else’s burning green*. Contrasts need not be maximally specific to highlight which feature of an event we want explained.

14 Schaffer (2005: 350, 2013: 56) in discussing how context shapes the relevant contrasts for causal claims, notes that some conversational contexts are not suitable to supply contrast cases for causal relations. These are cases where two events are assumed to have happened, and the contextually salient question is whether one caused the other. Because both events are assumed to have happened, there are no alternative live possibilities in the context set. We know the king ate rotten fruit and died. Did eating the fruit kill him? Here, there is no contextually salient possibility that he did not eat the fruit or lived to supply the relevant contrast. While there may be a worry for Schaffer, there is no worry for us. The question is a higher-order one, but it is still contrastive. The question is whether the (known) fact that the king ate the fruit rather than not eating it *explains* the (known) fact that he died rather than lived, *rather than not explaining it*. The contrasts are higher-order explanatory facts rather than first-order facts about the occurance of events.

15 A similar agent, the Serious Actualist, can be found in Roberts (2008). The difference between the Serious Actualist and the Actual Beer-Interested Agent is twofold: the Actual Beer-Interested Agent wants beer, not truth; she only wants truth if it gets her beer. And the Serious Actualist turns out to be concerned not only about believing actual truths, but reliably believing them. The Actual Beer-Interested Agent is happy with lucky beers; explaining her interest in reliability is, I think, part and parcel to the task of explaining her interest in modality generally.

16 I don’t mean to claim here that I can’t have desires with purely modal content; I might very well want people in my society to be able to freely choose which beer to drink, and think that this desire is just a desire that, possibly, they choose to drink beer $\alpha$ and as a result do drink beer $\alpha$. What I’m pointing out here is instead that, in many cases, the content of our desires is *not* modal, but nonetheless modal facts seem relevant to achieving those desires. Even when I only want actual beer, nonactual beers creep in to my decision making. Our account of nomic modality shouldn’t leave this mysterious.

17 At this point in my Humean story, I’ve appealed directly to the modal notion of manipulation. To give a fully compelling Humean account I would either need to show how this can be cashed out in Humean-friendly terms or argue that this apparent circularity shouldn’t bother us. But I don’t have the space to do that here, since I’m addressing a completely different supposed circularity. I point my readers to those papers mentioned above if they wish to see how this project is going.

18 Ruben (1990: 108–109) provides a similar role for laws but with a linguistic cast: “the laws provide the properties for determining under which descriptions a particular fully explains another. Laws provide the appropriate vocabulary for full singular explanation.”

19 Note that this contrastivity principle can escape some of the counterexamples to transitivity from Hicks and van Elswyk (2015), and that the response here does not require us to distinguish different sorts of explanation as in Loewer (2012).

20 Although elsewhere in his 2016 paper Lange responds to Hicks and van Elswyk (2015) by accepting that the correct transitivity principle is contrastive, he does not supply the contrasts in this metaexplanation. This is because he takes these objections to his argument to be distinct. In this paper I show how they are related.

21 Here I’ve suppressed the contrasts in the embedded explanation. The full explanatory fact is something like “The fact that *if* *c* *then* *e* *and if* $c'$ *then* $e'$ *are laws* rather than *both accidents* explains the fact that *c* *rather than* $c'$ *explains* *e* *rather than* $e'$ rather than *not explaining* *e* *rather than* $e'$.” In the next footnote I’ll point out why this doesn’t matter for my argument.

22 A similar response on behalf of the Humean is advanced by Dennison (2013) and discussed in Roberts (MS). Dennison argues on the basis of difference-making considerations that the occurance of *e* is not part of the explanation of *if c then e*’s lawhood on the basis that it is not a difference-maker in the sense of Strevens (2008).

23 Here we see why the embedded contrasts do not matter. For if *c* and *e* are accidentally, rather than lawfully, connected, it will still be the case that *e* occurs *rather than* $e'$.

24 With the possible exception, strangely, of Lange’s own view (in Lange 2009), according to which counterfactuals are basic and so unexplained.

25 A real explanation of this would be to show that Newtonian gravitation is a low-energy approximation of General Relativity. Such an explanation would both include general facts justifying the relevant simplifying assumptions and the laws of GR; on the present view the gravitational attraction would thereby be explained by the non-lawful facts showing why the simplifying assumptions hold, and the laws of GR would show why those facts explain gravitational attraction. But for the purpose of this example, let’s pretend that Newtonian physics is fundamental, or at least true.

26 There is something similar here in the case of null scientific explanation: not every explanandum without explanans will be null scientifically explained. For example logical and mathematical truths follow from the empty set of premises, but because no law is required for this inference, they are not null explained.
